# The Prognostic Role of Metabolic and Endocrine Parameters for the Clinical Severity of COVID-19

**DOI:** 10.1155/2022/5106342

**Published:** 2022-01-20

**Authors:** Shokoufeh Bonakdaran, Parvin Layegh, Solmaz Hasani, Mozhgan Afkhamizadeh, Zahra Mazloum Khorasani, Masoud Mohebbi, Shohreh Vojouhi, Zohreh Mousavi, Robab Beigom Aboutorabi, Hassan Mehrad-Majd, Amirhossein Sahebkar, Mohammad Ali Yaghoubi

**Affiliations:** ^1^Metabolic Syndrome Research Center, Mashhad University of Medical Sciences, Mashhad, Iran; ^2^Endocrine Research Center, School of Medicine, Mashhad University of Medical Sciences, Mashhad, Iran; ^3^Cancer Molecular Pathology Research Center, Mashhad University of Medical Sciences, Mashhad, Iran; ^4^Applied Biomedical Research Center, Mashhad University of Medical Sciences, Iran; ^5^Biotechnology Research Center, Pharmaceutical Technology Institute, Mashhad University of Medical Sciences, Mashhad, Iran; ^6^Department of Biotechnology, School of Pharmacy, Mashhad University of Medical Sciences, Mashhad, Iran

## Abstract

**Objective:**

An outbreak of coronavirus disease-19 (COVID-19) began in December 2019 and spread globally, overwhelming the entire world. COVID-19 is a public health emergency of international concern. Due to its high morbidity and mortality rate, recognition of its risk and prognostic factors is important. We aimed to understand the relationship between metabolic and endocrine parameters and the prognosis of COVID-19.

**Methods and Materials:**

This was a cross-sectional clinical study. A total of 70 patients with severe COVID-19 were enrolled. Laboratory results at the first admission time (including complete blood count, C-reactive protein, lactate dehydrogenase, blood glucose, calcium, phosphate, albumin, creatinine, magnesium, lipid profiles, liver enzymes, thyroid hormones, cortisol, and vitamin D) and outcome data were recorded. We divided patients into (1) intensive care unit- (ICU-) admitted and non-ICU-admitted and (2) survivors and nonsurvivors for estimation of severity and prognosis. We determined the risk factors associated with critical illness and poor prognosis.

**Results:**

Patients with higher white blood cell (WBC) count and phosphate levels had significantly higher ICU admission rates. According to univariate analysis, serum levels of T3, phosphate, and WBC as well as the duration of hospitalization were associated with mortality. Multivariate analysis revealed that only WBC and duration of hospitalization were independent predictors for mortality rate in COVID-19 patients.

**Conclusion:**

Our findings suggest that longer duration of hospitalization and higher WBC count are associated with poor outcomes in patients with COVID-19.

## 1. Introduction

The coronavirus disease-19 (COVID-19) pandemic in 2020 was a global crisis associated with high morbidity and mortality, especially in people with underlying diseases. It has been proposed that various comorbidities such as hypertension, diabetes mellitus (DM), obesity, cardiovascular disease (CVD), chronic respiratory disease, malignancies, and glucocorticoid use are among the most common chronic underlying disorders that predispose to and exacerbate COVID-19 infection [1, 2]. Identifying the COVID-19-associated risk factors may have a significant impact on the prevention and management of the disease and can ultimately reduce mortality in these patients.

It is likely that endocrine parameters may play a role in the prognosis of COVID-19 infection as well. Among the predisposing factors for COVID-19, DM has been reported as one of the most common risk factors [3–5]. It has also been found that the presence of DM is a major risk factor for the severity and poor prognosis of the disease because it confers an impaired immunologic system [6–8]. Hypoglycemia can occur in patients with COVID-19, and even in nondiabetic patients, blood glucose should be closely monitored [9, 10]. Hepatic and renal involvement drugs such as hydroxychloroquine and sepsis can cause adrenal damage or functional adrenal insufficiency, which may cause hypoglycemia and affect prognosis [11]. It has been supposed that mineral and vitamin deficiencies, including vitamin D deficiency, affect the incidence and severity of COVID-19 [12]. Functional adrenal insufficiency is possible during any severe disease and in patients treated with glucocorticoids due to primary and secondary adrenal insufficiency [13]. Measuring the adrenal response seems to be helpful in determining the severity and prognosis of COVID-19 disease.

Among the systemic complications and manifestations of viral infections, subacute thyroiditis is not uncommon [14]. Previous studies of thyroid function tests in hospitalized patients with COVID-19 infection have shown a decrease in TSH and total T3 levels, which were directly related to the severity of the disease [15].

In patients with COVID-19, like many other acute illnesses, calcium levels can be reduced due to concomitant albumin and magnesium deficiency for a variety of reasons, such as poor nutrition or the effects of cytokines on parathyroid function [16]. Previous studies suggest that calcium levels can be used as a marker to determine the severity of COVID-19 disease [17].

Given the aforementioned abnormalities in COVID-19, this study was designed to identify common endocrine changes in severe COVID-19 and the effect of these variables on the patient's outcome.

## 2. Methods

This cross-sectional study was performed on 120 patients with severe COVID-19 infection. Patients were selected after referring to the emergency department of the Imam Reza Hospital in Mashhad, Iran. Written informed consent was obtained from each patient with an indication for hospitalization, and the study was conducted in accordance with the protocol approved by the Ethics Committee of the Mashhad University of Medical Sciences. Around 5 cc of venous blood samples was taken from each patient before initiating treatment and was immediately sent for serum measurements to the laboratory.

Patients with strong clinical signs and symptoms of COVID-19 whose diagnoses were confirmed by lung computed tomography (CT) scan or positive results from RT-PCR testing from nasopharyngeal swab sample tests were enrolled in the study. Patients with a history of glucocorticoid use before sampling, thyroid diseases, taking drugs that affect thyroid hormone levels (dopamine, lithium, interferons, and tyrosine kinase inhibitors) or conditions that affect calcium and vitamin D levels were excluded from this study.

Serum levels of biochemical parameters, including blood glucose, lipid profile, calcium (Ca), phosphate (P), albumin, magnesium, sodium, potassium, liver function tests, creatinine, (WBC), lymphocyte count, erythrocyte sedimentation rate (ESR), high sensitivity C-reactive protein (hs-CRP), lactate dehydrogenase (LDH), free T4, total T3 and TSH, cortisol, and vitamin D were measured using the routine methods. Moreover, all patients were classified according to the vitamin D levels into 3 groups of deficiency (<20 ng/ml), insufficiency (>20- <30 ng/ml), and sufficiency (>30 ng/ml). Most biochemical tests were conducted based on enzymatic methods. Serum levels of Ca, P, albumin, magnesium, sodium, and potassium were measured using atomic absorption spectrometry.

Patients were divided into two groups considering their outcome of recovery or death; they were also classified in two groups according to the disease severity: intensive care unit- (ICU-) admitted and non-ICU-admitted. Patients with relative improvement were followed up to three months after discharge and evaluated for disease outcome and readmission, if needed. Clinical and laboratory variables of patients were compared in the aforementioned groups.

Statistical analysis was performed using SPSS Version 22.0 statistics software package. The results were presented as mean ± SD or median (interquartile range) for data with normal and non-normal distributions, respectively. Qualitative variables were expressed as percentage. Normality of distribution for the quantitative data was checked using the Kolmogorov-Smirnov test. Student's *t*-test was used for variables with normal distribution and quantitative variables with nonnormal distribution were compared with Mann–Whitney. Qualitative variables between the groups were analyzed by chi-square or Fisher exact tests. The level of significance was set at 0.05.

## 3. Results

Out of 120 selected patients whose serum samples were taken, the information of 70 patients was analyzed. Fifty patients were excluded from the study after discharge due to lack of access to accurate documents during hospitalization and lack of feedback from the patients. Demographic and laboratory information at the time of admission of patients is specified in Table 1.

Among the patients, 43.9% had a positive history of diabetes, 35% had a history of hypertension, and 18.4% had a positive history in favor of cardiovascular problems. Of the total 70 patients, 39 (55.7%) were male. The studied parameters in the two sexes were compared with each other, and the results are summarized in Table 2. No significant difference in 90 days mortality rate was found for gender distribution (38.2% in men and 42.3% in women (*P* = 0.750)).

As shown in Table 2, only the level of vitamin D in hospitalized women was significantly higher than hospitalized men (*P* = 0.046) and other parameters were not significantly different between the genders (*P* > 0.05). Among the patients, 59.6% required ICU admission. A comparative study of the studied parameters between ICU-admitted and non-ICU-admitted patients is presented in Table 3. Among patients who had ICU admission, the duration of hospitalization (*P* < 0.001), WBC count (*P* = 0.02), phosphate levels (*P* = 0.01), readmission (*P* = 0.036), and mortality rate (*P* < 0.0001) were significantly higher than the non-ICU-admitted group whereas other variables were not significantly different between the two groups.

As mentioned, 38.2% of infected men and 42.3% of infected women died (24 patients in total). The characteristics of patients with these two different outcomes (survivors or nonsurvivors) are given in Table 4. As shown in this table, according to the outcome, there was a longer hospitalization duration (*P* = 0.002), higher WBC count (*P* < 0.001), higher phosphate (*P* = 0.04), and lower serum T3 levels (*P* = 0.03) in the death group. In addition, comorbidities like DM, HTN, and CVD showed nonsignificantly higher frequency in nonsurvivors compared to survivors.

To determine the independent effects of hospitalization duration, WBC count, serum phosphate, and T3 levels, univariate and multivariate logistic regression analyses were performed. The results showed that both duration (OR = 1.11, 95%CI = 1.01–1.22, *P* = 0.025) and WBC (OR = 1.22, 95%CI = 1.01–1.64, *P* = 0.042) were independent predictors of mortality in patients with COVID-19.

Stratified analysis based on vitamin D levels revealed no significant differences in ICU admission between patients with and without vitamin D deficiency (60.7% vs. 60.0%, *P* = 0.960). However, the mortality rate was found to be nonsignificantly higher in vitamin D-deficient patients compared to the vitamin D-sufficient group (40.0% vs. 60.9%, *P* = 0.120).

**Figure 1:**
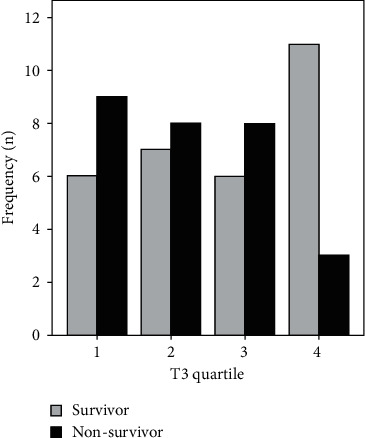
Patients' outcomes based on T3 quartiles.

The levels of serum T3 were divided into four quartiles, the corresponding graph of which is shown in Figure 1. A nonsignificant negative association can be seen between T3 concentrations and the mortality rate (*P* = 0.283).

## 4. Discussion

This study aimed to evaluate and report endocrine parameters and related outcomes in an Iranian population of COVID-19 patients. In this study, serum levels of T3, phosphate, WBC, and duration of hospitalization were associated with mortality in infected patients.

In the COVID-19 pandemic, diabetes is considered one of the most common diseases associated with increased morbidity and mortality. In a meta-analysis of the relationship between diabetes and COVID-19 disease, the prevalence of diabetes in COVID-19 patients in China was 12.6%, in Italy 34.7% and in the United States, 34.4% [3]. In a study in Iran, 595 patients with COVID-19 were studied, and 148 (24.9%) had diabetes [18]. In our study, among 70 hospitalized patients, 43.9% had a positive history for diabetes, which was more than what is reported in other studies.

In some studies, the relationship between hyperglycemia and increased mortality in both diabetic and nondiabetic patients with COVID-19 has been suggested as an independent factor [19], while in the present study, there was no significant difference in blood sugar levels between patients admitted to the ICU and normal wards (*P* = 0.81). Although the median blood glucose was higher in patients with mortality than those who improved, this difference was not statistically significant (*P* = 0.44). In the study by Polverino et al., diabetes was not significantly associated with the risk of death in COVID-19 patients [20]. In the study of Bellan et al., the percentage of diabetic patients in COVID-19 patients who were discharged and died did not show a significant difference [21].

Coronavirus infection appears to cause changes in lipid profile. According to studies in this field, the levels of total cholesterol, LDL cholesterol, and HDL cholesterol are reduced during infection [22]. Changes in liver function during the disease can reduce lipid synthesis. Moreover, increased release of tumor necrosis factor alpha (TNF*α*), interferon-gamma (INF-*γ*), and even interleukins produced during infection reduce the synthesis and secretion of lipoproteins, as well as alteration in the distribution of lipoproteins from intravascular to extravascular space [23]. In our study, although many of the included patients had diabetes or other metabolic syndrome features and samples were taken during the nonfasting period, the average lipid levels were low and no significant difference was observed between survivors and nonsurvivors. Contrary to our study, in some studies, lower levels of HDL cholesterol have been associated with higher mortality and are considered a predictor of disease severity [24]. Further studies are needed to clarify this discrepancy.

In the current study, increased leukocyte count was recognized as a predictor for disease severity, indicating an increased response to systemic inflammation and subsequent cytokine storms and tissue damage. In the study by Corradini et al., WBC was positively associated, and lymphocyte count was negatively associated with the risk of death [25]. In the study by Bellan et al., the number of WBCs and neutrophils in deceased patients was significantly higher than those discharged, while the number of lymphocytes in deceased patients was significantly lower [26]. The results of these studies are consistent with the present study. However, some studies have confirmed the role of lymphopenia as a marker of mortality in these patients [27, 28], but it was not proven in the present study.

The role of vitamin D in adaptive immunity has been proven. It also stimulates cellular immunity through induction of antimicrobial peptides. This vitamin appears to inhibit cytokine storm by reducing inflammatory cytokines such as TNF-*α* and INF-*γ* [29]. In our study, there was no significant difference between the mean of serum vitamin D levels in the ICU group and the non-ICU-admitted patients. In line with our results, in *Panagiotou* study, vitamin D levels did not differ between the two groups, but the percentage of patients with vitamin D less than 20 ng/ml was higher in the ICU-admitted group [29]. In the *Pizzini* study in Austria, vitamin D level was not associated with disease severity [30]. In our study, mean vitamin D levels were higher in patients who died, although this difference was not significant. On the other hand, the level of vitamin D in our study was higher in women, and perhaps taking more supplements justifies this condition. In *Hastie* studies with high sample sizes, vitamin D level was not associated with mortality in patients with COVID-19 [31]. However, in other studies, low levels of vitamin D levels were inversely related to severity and mortality [32–35].

Higher serum concentrations of phosphate in our analysis, even within the normal range, were associated with an increased risk of severity and mortality. To the best of our knowledge, this association with COVID-19 mortality is novel. Phosphate has various physiological effects. It is necessary for respiratory muscle function, supplying of oxygen to tissues, intracellular metabolism, and electrolyte homeostasis. Furthermore, it has a role in the coagulation cascade as well as the body immune system.

Phosphate levels, which have been linked to increased mortality, are regulated physiologically by a complex interplay among 1,25-dihydroxyvitamin D_3_, PTH, and FGF23 [36]. Although in our study PTH and FGF23 levels were not determined, adjustments for vitamin D levels did not modify our results.

One study has resulted that phosphate induce injury in lung epithelial cells through increased oxidative stress and apoptosis [37]. Several studies in this regard resulted that higher levels of phopahate level is associated with an increased risk of mortality in sepsis, cardiovascular disease and COPD patients [36, 38–40] that is similar with our results.

Similar to previous studies, a high percentage of our patients had low levels of T3 and was associated with a high mortality rate [41–43]. This alteration in thyroid function in severe disease results from changes in the expression of some genes involved in the metabolism of thyroid hormones [44]. Cortisol is a biomarker that is expected to be higher in patients with severe COVID-19 involvement due to the stress response and inflammatory processes. The effect of COVID-19 infection on cortisol levels is still clearly unknown. Pal et al. showed that serum cortisol level is an independent factor predicting disease severity and mortality in hospitalized patients with community-acquired pneumonia [45]. In our study, the level of cortisol in critically ill patients admitted to ICU was higher than that of other patients admitted to general COVID-19 departments, but this difference was not significant statistically (*P* = 0.12). Since there is between-subject variability in cortisol response to stress, this biomarker is not commonly used in determining the prognosis of patients. There are limitations in interpreting the relationship between cortisol levels and mortality rate in our study as well as other studies. The pulsatile and circadian nature of cortisol secretion interprets the single cortisol sample taken at the time of admission. Besides, due to serum albumin changes which affect directly the total serum cortisol levels, and simultaneous hypoalbuminemia which was present in most of our admitted patients, the total cortisol levels in our study could not reflect the actual cortisol levels in these patients, and perhaps the estimation of free hormone levels or serial measurement of cortisol would change the results.

There are several limitations, which might have caused potential bias in our research. The study was single centered, with limited sample size. Moreover, our results were based on the first laboratory data of patients and not the dynamic changes monitored. Lack of long-term monitoring on postdischarge complications and outcomes is another limitation that needs to be addressed in future studies.

In conclusion, on the basis of the current results, longer duration of hospitalization and higher WBC count are associated with poor prognosis in COVID-19 patients.

## Figures and Tables

**Table 1:** Demographic and laboratory data of all subjects.

Variable	Mean ± SD/median (IQR)
Age (years)	61.0 ± 15.3
Duration of hospitalization (days)	14.5 ± 13.3
WBC count	7800 (6200-10100)
Lymphocyte count	1142.2 ± 782.9
CRP (mg/lit)	32.15 (17.75-90.98)
ESR (mm/h)	52.6 ± 35.7
LDH (U/l)	570.0 (374.25-822.25)
BS (mg/dl)	155.0 ± 85.6
Cholesterol (mg/dl)	131.9 ± 47.8
TG (mg/dl)	108.0 ± 63.6
HDL (mg/dl)	37.1 ± 10.6
LDL (mg/dl)	63.4 ± 24.9
Ca (mg/dl)	8.9 ± 0.8
P (mg/dl)	3.7 ± 1.4
Albumin (gr/dl)	2.7 ± 0.6
Mg (mg/dl)	3.0 ± 0.6
ALT (IU/l)	26.5 (15.25-51.50)
AST (IU/l)	38.00 (25.00-65.00)
Na (mEq/l)	135.6 ± 4.5
K (mmol/l)	4.3 ± 0.8
Cr (mg/dl)	1.5 ± 1.5
T3 (ng/dl)	48.1 ± 23.6
Free T4 (ng/dl)	1.0 ± 0.4
TSH (mIU/l)	1.00 (0.37-1.99)
Cortisol (*μ*g/dl)	39.80 (11.58-51.15)
Vitamin D (ng/ml)	16.25 (8.1-28.6)

IQR: interquartile range; WBC: white blood cell; ESR: erythrocyte sedimentation rate; CRP: C-reactive protein; LDH: lactate dehydrogenase; BS: blood sugar; TG: triglyceride: LDL: low-density lipoprotein; HDL: high-density lipoprotein; ALT: alanine aminotransferase; AST: aspartate aminotransferase; Ca: calcium; Mg: magnesium; Cr: creatinine; Na: natrium; K: kalium; T3: tri-iodotironina; TSH: thyroid-stimulating hormone; 25 OHD: 25-hydroxy vitamin D; P: phosphate.

**Table 2:** Laboratory parameters according to gender.

Variable	Male (mean ± Sd)/median (IQR)	Female (mean ± Sd)/median (IQR)	*P* value
Age (years)	59.6 ± 15.2	62.6 ± 15.6	0.382
Duration of hospitalization (days)	12.2 ± 8.6	17.4 ± 17.3	0.371
WBC	7200 (6300-10000)	8000 (5900-14050)	0.654^∗^
Lymphocyte	1090.1 ± 836.4	1206.4 ± 720.5	0.394
ESR (mm/h)	50.0 ± 32.8	56.4 ± 40.4	0.093
CRP (mg/lit)	28.0 (12.25-160.33)	38.65 (36.6-69.0)	0.761^∗^
LDH (U/l)	657 (359-869.5)	498 (366-690)	0.347^∗^
BS (mg/dl)	155.3 ± 95.6	154.6 ± 71.0	0.622
Cholesterol (mg/dl)	134.3 ± 56.2	128.7 ± 33.8	0.891
LDL (mg/dl)	62.8 ± 24.3	64.2 ± 26.4	0.843
TG (mg/dl)	105.0 ± 69.2	112.0 ± 56.2	0.221
HDL (mg/dl)	38.8 ± 12.4	35.0 ± 7.4	0.393
ALT (IU/l)	31.5 (17.5-47.0)	24.5 (12.25-58.50)	0.613^∗^
AST (IU/l)	39 (25-67.25)	36 (24.5-65.5)	0.953^∗^
Ca (mg/dl)	9.0 ± 0.7	8.9 ± 0.9	0.682
P (mg/dl)	3.5 ± 1.4	4.0 ± 1.4	0.521
Albumin (gr/dl)	2.8 ± 0.5	2.6 ± 0.7	0.110
Mg (mg/dl)	3.0 ± 0.7	3.0 ± 0.55	0.181
Cr (mg/dl)	1.4 ± 1.2	1.7 ± 1.9	0.622
Na (mEq/l)	135.5 ± 4.0	135.8 ± 5.3	0.823
K (mmol/l)	4.3 ± 0.5	4.4 ± 1.2	0.894
T3 (ng/dl)	49.8 ± 22.3	45.7 ± 25.7	0.414
Free T4 (ng/dl)	1.0 ± 0.4	0.9 ± 0.4	0.993
TSH (mIU/l)	0.57 (0.31-1.75)	1.17 (0.61-2.59)	0.074^∗^
Cortisol (*μ*g/dl)	34.11 (9.59-51.15)	41.3 (19.25-57.40)	0.316^∗^
Vitamin D (ng/ml)	12.95 (8.07-24.68)	24.5 (9.98-38.63)	0.046^∗^
Mortality (%)	13 (38.2)	11 (42.3)	0.750

IQR: interquartile range; WBC: white blood cell; ESR: erythrocyte sedimentation rate; CRP: C-reactive protein; LDH: lactate dehydrogenase; BS: blood sugar; TG: triglyceride; LDL: low-density lipoprotein; HDL: high-density lipoprotein; ALT: alanine aminotransferase; AST: aspartate aminotransferase; Ca: calcium; Mg: magnesium; Cr: creatinine; Na: natrium; K: kalium; T3: tri-iodotironina; TSH: thyroid-stimulating hormone; P: phosphate. ^∗^*P* value of nonparametric Mann–Whitney *U* test.

**Table 3:** Comparison of parameters between ICU-admitted and non-ICU-admitted patients.

Variable	Non-ICU	ICU	*P* value
Age (years)	62.6 ± 14.9	62.6 ± 14.2	0.83
Duration of hospitalization (days)	8.39 ± 5.8	19.7 ± 16.3	0.01
WBC	6500 (5000-8700)	8000 (6675-11825)	0.02^∗^
Lymphocyte	1161.4 ± 653.9	1218.4 ± 925.5	0.76
ESR (mm/h)	47.1 ± 30.7	53.7 ± 38.1	0.09
CRP (mg/lit)	28 (11.75-119.0)	45 (25-96.65)	0.63^∗^
LDH (U/l)	530 (335.75-718.75)	586 (418.5-1123.8)	0.28^∗^
BS (mg/dl)	117.5 ± 72.3	159.6 ± 101.0	0.81
Cholesterol (mg/dl)	132.4 ± 26.8	136.2 ± 60.8	0.48
LDL (mg/dl)	64.0 ± 22.0	64.4 ± 27.2	0.25
TG (mg/dl)	109.0 ± 61.6	111.2 ± 72.3	0.98
HDL (mg/dl)	38.1 ± 10.3	38.1 ± 11.6	0.76
ALT (IU/l)	22.5 (11.5-42.0)	34 (16-94)	0.10^∗^
AST (IU/l)	37.5 (23.5-54.5)	39 (25-87.5)	0.26^∗^
Ca (mg/dl)	9.0 ± 0.8	8.9 ± 0.8	0.85
P (mg/dl)	3.6 ± 1.0	3.9 ± 1.6	0.01
Albumin (gr/dl)	2.7 ± 0.8	2.6 ± 0.5	0.58
Mg (mg/dl)	3.0 ± 0.6	3.0 ± 0.6	0.95
Cr (mg/dl)	1.5 ± 1.6	1.7 ± 1.7	0.47
Na (mEq/l)	135.5 ± 3.8	135.5 ± 5.2	0.96
K (mmol/l)	4.3 ± 0.4	4.3 ± 0.9	0.95
T3 (ng/dl)	56.1 ± 27.5	43.4 ± 20.7	0.11
Free T4 (ng/dl)	0.97 ± 0.52	0.97 ± 0.4	0.48
TSH (mIU/l)	1.12 (0.56-1.99)	0.84 (0.36-2.55)	0.52^∗^
Cortisol (*μ*g/dl)	36.54 (11.6-45.3)	42.9 (13.1-67.2)	0.12^∗^
Vitamin D (ng/ml)	8.7 (8.0-32.5)	19.3 (8.8-33.3)	0.42^∗^
Diabetes (%)	7 (30.4)	10 (29.4)	0.934^∗∗^
Smoking (%)	5 (21.7)	3 (8.8)	0.247^∗∗∗^
CVD (%)	3 (13.0)	3 (8.8)	0.677^∗∗∗^
Hypertension (%)	2 (8.7)	10 (29.4)	0.097^∗∗∗^
Readmission (%)	1 (4.5)	9 (26.5)	0.036^∗∗∗^
Mortality (%)	0 (0)	24 (70.6)	<0.0001

IQR: interquartile range; WBC: white blood cell; ESR: erythrocyte sedimentation rate; CRP: C-reactive protein; LDH: lactate dehydrogenase; BS: blood sugar; TG: triglyceride; LDL: low-density lipoprotein; HDL: high-density lipoprotein; ALT: alanine aminotransferase; AST: aspartate aminotransferase; Ca: calcium; Mg: magnesium; Cr: creatinine; Na: natrium; K: kalium; T3: tri-iodotironina; TSH: thyroid-stimulating hormone; CVD: cardiovascular diseases; P: phosphate. ^∗^*P* value of nonparametric Mann–Whitney *U* test. ^∗∗^*P* value of nonparametric *Q*-square test. ^∗∗∗^*P* value of the Fisher exact test.

**Table 4:** Comparison of different variables between two groups of survivors and nonsurvivors.

Variable	Survivors mean ± SD or ^∗^median (IRQ)	Nonsurvivors mean ± SD or ^∗^median (IRQ)	*P* value
Age	60.9 ± 15.0	61.0 ± 15.8	0.98
Duration of hospitalization (days)	10.17 ± 7.60	21.29 ± 18.43	0.002
WBC	6650 (5225-8600)	8900 (7025-13700)	<0.001^∗^
Lymphocyte	1017.1 ± 607.8	1391.3 ± 1019.6	0.36
ESR (mm/h)	51.5 ± 35.4	47.8 ± 36.5	0.82
CRP (mg/lit)	31.0 (14.5-71.0)	47.5 (25.0-98.0)	0.70^∗^
LDH (U/l)	611.9 ± 381.6	802.3 ± 533.2	0.21
BS (mg/dl)	143 ± 64.69	167.3 ± 117.2	0.44
Cholesterol (mg/dl)	123.1 ± 30.5	141.1 ± 59.9	0.21
LDL cholesterol (mg/dl)	60.4 ± 22.9	66.4 ± 26.9	0.35
TG (mg/dl)	95.7 ± 53.9	125.4 ± 79.4	0.06
HDL cholesterol (mg/dl)	36.10 ± 10.1	39.28 ± 12.35	0.38
ALT (IU/l)	25.0 (12.5-44)	31.5 (15.5-132.5)	0.19^∗^
AST (IU/l)	36.0 (23-63.5)	39.0 (25-73)	0.22^∗^
Ca (mg/dl)	8.8 ± 0.8	9.1 ± 0.7	0.24
P (mg/dl)	3.4 ± 1.2	4.1 ± 1.6	0.04
Albumin	2.7 ± 0.65	2.7 ± 0.62	0.94
Mg (mg/dl)	3.0 ± 0.67	3.0 ± 0.63	0.93
Cr (mg/dl)	0.9 (0.8-1.10)	1.2 (0.9-1.93)	0.17^∗^
Na (mEq/l)	135.6 ± 3.9	135.7 ± 5.2	0.86
K (mmol/l)	4.3 ± 0.5	4.4 ± 1.2	0.98
T3 (ng/dl)	54.9 ± 25.8	42.2 ± 21.4	0.03
Free T4 (ng/dl)	1.01 ± 0.45	0.91 ± 0.41	0.88
TSH (mIU/l)	1.17 (0.56-2.07)	0.58 (0.32-2.55)	0.13^∗^
Cortisol (*μ*g/dl)	38.7 (15.29-51.15)	42.9 (13.05-64.10)	0.86^∗^
Vitamin D (ng/ml)	13.6 (8.0-26.30)	22.7 (10.65-42.30)	0.07^∗^
Vitamin D deficiency	21 (70)	14 (50)	0.120
Smoking (%)	6 (16.7)	2 (5.9)	0.261^∗∗∗^
Hypertension (%)	8 (22.2)	6 (17.6)	0.632^∗∗^
Diabetes (%)	11 (30.6)	7 (20.6)	0.304^∗∗∗^
CVD (%)	3 (8.3)	4 (11.8)	0.706^∗∗∗^

IQR: interquartile range; WBC: white blood cell; ESR: rrythrocyte sedimentation rate; CRP: C-reactive protein; LDH: lactate dehydrogenase; BS: blood sugar; TG: triglyceride; LDL: low-density lipoprotein; HDL: high density lipoprotein; ALT: alanine aminotransferase; AST: aspartate aminotransferase; Ca: calcium; Mg: magnesium; Cr: creatinine; Na: natrium; K: kalium; T3: tri-iodotironina; TSH: thyroid-stimulating hormone; CVD: cardiovascular diseases; P: phosphate. ^∗^*P* value of nonparametric Mann–Whitney *U* test. ^∗∗^*P* value of nonparametric *Q*-square test. ^∗∗∗^*P* value of The Fisher exact test.

## Data Availability

There is no raw data associated with this review article.
